# Competing-risk analysis of coronavirus disease 2019 in-hospital mortality in a Northern Italian centre from SMAtteo COvid19 REgistry (SMACORE)

**DOI:** 10.1038/s41598-020-80679-2

**Published:** 2021-01-13

**Authors:** Valentina Zuccaro, Ciro Celsa, Margherita Sambo, Salvatore Battaglia, Paolo Sacchi, Simona Biscarini, Pietro Valsecchi, Teresa Chiara Pieri, Ilaria Gallazzi, Marta Colaneri, Michele Sachs, Silvia Roda, Erika Asperges, Matteo Lupi, Alessandro Di Filippo, Elena Seminari, Angela Di Matteo, Stefano Novati, Laura Maiocchi, Marco Enea, Massimo Attanasio, Calogero Cammà, Raffaele Bruno

**Affiliations:** 1 U.O.C. Malattie Infettive I Fondazione IRCCS Policlinico San Matteo, Università di Pavia, Pavia, Italy; 2 Section of Gastroenterology and Hepatology, Department of Health Promotion, Mother and Child Care, Internal Medicine and Medical Specialties, University of Palermo, Palermo, Italy; 3 Doctoral Programme in Oncology and Experimental Surgery, Department of Surgical, Oncological and Stomatological Disciplines, University of Palermo, Palermo, Italy; 4 Department of Economics, Business and Statistics, University of Palermo, Palermo, Italy; 5 Department of Health Promotion, Mother and Child Care, Internal Medicine and Medical Specialties, University of Palermo, Palermo, Italy; 6 Department of Clinical, Surgical, Diagnostic, and Pediatric Sciences, University of Pavia, Pavia, Italy

**Keywords:** Diseases, Infectious diseases, Viral infection

## Abstract

An accurate prediction of the clinical outcomes of European patients requiring hospitalisation for Coronavirus Disease 2019 (COVID-19) is lacking. The aim of the study is to identify predictors of in-hospital mortality and discharge in a cohort of Lombardy patients with COVID-19. All consecutive hospitalised patients from February 21st to March 30th, 2020, with confirmed COVID-19 from the IRCCS Policlinico San Matteo, Pavia, Lombardy, Italy, were included. In-hospital mortality and discharge were evaluated by competing risk analysis. The Fine and Gray model was fitted in order to estimate the effect of covariates on the cumulative incidence functions (CIFs) for in-hospital mortality and discharge. 426 adult patients [median age 68 (IQR 56 to 77 years)] were admitted with confirmed COVID-19 over a 5-week period; 292 (69%) were male. By 21 April 2020, 141 (33%) of these patients had died, 239 (56%) patients had been discharged and 46 (11%) were still hospitalised. Among these 46 patients, updated as of 30 May, 2020, 5 (10.9%) had died, 8 (17.4%) were still in ICU, 12 (26.1%) were transferred to lower intensity care units and 21 (45.7%) were discharged. Regression on the CIFs for in-hospital mortality showed that older age, male sex, number of comorbidities and hospital admission after March 4th were independent risk factors associated with in-hospital mortality. Older age, male sex and number of comorbidities definitively predicted in-hospital mortality in hospitalised patients with COVID-19.

## Introduction

Since December 2019 SARS COV 2 disease, defined as a pandemic by the World Health Organization (WHO) on 11 March 2020, has spread rapidly all over the world^1^. Outside China, the first western country to be affected was Italy, where the epidemic began on 21 February 2020 and quickly affected thousands of people, practically overwhelming the capacity of the National Health System to respond to it in terms of availability of hospital, ICU beds and ER spaces to receive and manage patients^2^. Although, Policlinico San Matteo is one of the largest teaching hospitals (1300 beds) in the region and the Infectious Diseases division managed to more than double its total capacity of regular beds from 44 to 94, in the first 2 weeks it experienced difficult in allocating patients, because clinical criteria to define the evolution of the disease were, and still are, missing^3^.

To date, most of the studies that have extensively reported the clinical and laboratory characteristics of patients infected by COVID-19 have been carried out in China^4^. Data on clinical outcomes and treatment of COVID-19 outside China are lacking and the high heterogeneity in observed case-fatality ratios between and within different countries still remains unexplained. Because COVID-19 shows an array of clinical presentations and the lack of effective treatment makes it difficult to predict its outcome, the identification of risk factors for clinical outcomes, such as death, ICU admission and hospital discharge is crucial in order to improve the organisation of healthcare and to identify patients who may benefit the most from the available treatment strategies. Moreover, in such a complex epidemiological and clinical scenario, competing risks might help in the assessment of the impact of treatment strategies on meaningful clinical endpoints, such as in-hospital death and discharge^5^.

The aim of this study was to explore and explain, in a cohort of Lombardy patients with COVID-19 in Pavia, Italy, the heterogeneity of clinical outcomes and to identify predictors of in-hospital mortality and discharge by competing risks analysis.

## Results

From 22 February to 30 March 2020, 426 confirmed cases of COVID-19 were observed, 292 (68.5%) were males (Table 1). Median age was 68 years (IQR, 56 to 77 years) and 197 (45.8%) patients were older than 70 years of age. 269 (63%) patients had at least one comorbidity, with hypertension and diabetes being the most common (140 (33%) and 63 (15%) patients, respectively). The median score on the Charlson comorbidity index (CCI)^6^ was 3 (IQR, 1 to 4) while the median score of Modified Elixhauser score (mEI)^7^ was 9.2 ± 7.8. The first nasal swab test for SARS-COV2 was positive in 365 (86%) patients, while 61 (14%) patients had a negative first nasal swab test and positive repeat nasal swab test. Laboratory findings on admission are reported in Table 1. Lymphocytopenia was present in 398 (93.3%) patients, while platelet count was lower than 150,000/mmc in 100 (23.5%) patients. CRP was increased in 188 (44.0%) patients and LDH was elevated in 369 (87.0%) patients. Chest radiography revealed the presence of interstitial pneumonia in 301 (71.0%) patients.

**Table 1:** Demographic, clinical and laboratory characteristic of patients on admission.

	Overall (n = 426)	Death (n = 141)	Survivor (n = 285)	*p*-value
**Age (years)**	68.0 (56.0–77.0)	77.0 (71.0–83.0)	61.0 (50.0–72.0)	<0.0001
< 50	72 (16.9%)	1 (0.7%)	71 (24.9%)	<0.0001
50–59	64 (15.0%)	8 (5.7%)	56 (19.6%)	0.0003
60–69	95 (22.3%)	21 (14.9%)	74 (25.9%)	0.014
70–79	125 (29.3%)	66 (46.8%)	59 (20.7%)	<0.0001
> 80	70 (16.4%)	45 (31.9%)	25 (8.8%)	<0.0001
Male sex	292 (68.5%)	103 (73.0%)	189 (66.3%)	0.194
Comorbidity	269 (63.1%)	116 (82.2%)	153 (53.7%)	<0.0001
Hypertension	140 (32.8%)	52 (36.8%)	88 (30.9%)	0.256
Diabetes	63 (14.8%)	28 (19.9%)	35 (12.3%)	0.074
Atrial fibrillation	37 (8.7%)	21 (14.9%)	16 (5.6%)	0.002
Coronary heart disease	36 (8.5%)	25 (17.7%)	11 (3.9%)	<0.0001
Obesity	26 (6.1%)	10 (7.1%)	16 (5.6%)	0.636
Chronic kidney disease	25 (5.9%)	16 (11.3%)	9 (3.2%)	0.0007
Chronic heart failure	21 (4.9%)	12 (8.5%)	9 (3.2%)	0.027
Chronic liver disease	21 (4.9%)	11 (7.8%)	10 (3.5%)	0.085
Chronic obstructive lung disease	20 (4.7%)	9 (6.4%)	11 (3.9%)	0.342
History of malignancy	18 (4.2%)	4 (2.8%)	14 (4.9%)	0.467
Active malignancy	16 (3.8%)	8 (5.7%)	8 (2.8%)	0.182
Dementia	12 (2.8%)	9 (6.3%)	3 (1.1%)	0.005
Charlson comorbidity index	3 (1–4)	4 (3–5)	2 (1–3)	<0.0001
Modified Elixhauser score	9.2 ± 7.8	15.0 ± 7.9	6.4 ± 5.9	<0.0001
**Number of comorbidities**				<0.0001
0	155 (36.3%)	25 (17.7%)	130 (45.6%)	
1	145 (34.0%)	52 (36.9%)	93 (32.6%)	
2	73 (17.1%)	34 (24.1%)	39 (13.7%)	
More than 3	53 (12.4%)	31 (22.0%)	22 (7.7%)	
Median time from symptoms onset to hospitalization	7 (3–10)	6 (3–8)	8 (4–11)	0.037
**Time of hospital admission**				0.025
From February, 21 to March, 3	137 (32.2%)	36 (25.5%)	101 (35.4%)	
From March, 4 to March, 16	165 (38.7%)	67 (40.6%)	98 (34.3%)	
From March, 17 to March, 30	124 (29.1%)	38 (27.0%)	86 (30.2%)	
Glutamic oxaloacetic transaminase, U/L	41 (28–64)	44 (29–70)	40 (27–57)	0.117
Glutamic pyruvic transaminase, U/L	32 (21–48)	34 (23–53)	31 (21–44)	0.258
C-reactive protein, mg/dL	8.23 (4.14–14.75)	10.40 (5.85–15.00)	7.64 (3.62–14.54)	0.008
C-reactive protein>10 mg/dL	188 (44.1%)	83 (58.9%)	105 (36.8%)	<0.0001
Creatinine, mg/dL	0.89 (0.72–1.11)	0.90 (0.75–1.16)	0.87 (0.71–1.09)	0.132
Lactate dehydrogenase, U/L	365 (304–446)	380 (325–455)	365 (294–446)	0.075
Lactate dehydrogenase>245 U/L	369 (86.6%)	129 (91.5%)	240 (84.2%)	0.054
Troponine, ng/L	26 (10–108)	21 (10–55)	37 (11–119)	0.103
White cell blood count, × 10^9^ per L	6.73 (5.18–9.15)	7.02 (4.95–8.90)	6.65 (5.35–9.3)	0.423
Lymphocyte count, × 10^9^ per L	0.80 (0.60–1.00)	0.74 (0.60–0.97)	0.80 (0.60–1.01)	0.087
Lymphocyte count<1.5 × 10^9^ per l	398 (93.3%)	135 (95.7%)	263 (92.3%)	0.250
Neutrophil count, × 10^9^ per l	5.27 (3.9–7.72)	5.50 (3.61–7.68)	5.2 (3.94–7.75)	0.455
Platelet count, × 10^9^ per L	204 (152–287)	201 (144–263)	207 (154–296)	0.184
Platelet count < 150 × 10^9^ per L	100 (23.5%)	39 (27.7%)	61 (21.4%)	0.236
Pneumonia at chest X-ray	301 (70.7%)	130 (92.2%)	171 (60.0%)	<0.0001

Data are expressed as median (interquartile range) or n (%).

Data on treatments are reported in Table 2. Antibiotic therapy was started in 304 (85%) of patients. Corticosteroid treatment was administered to 70 (20%) patients and consisted of dexamethasone 20 mg daily in 13 patients and, starting on 21 March 2020, methylprednisolone 1 mg/kg intravenously daily in 57 patients. Hydroxycloroquine, 600 mg twice on day 1, then 400 mg daily for 7 days, was administered to 249 (70.3%) patients and was initiated within 72 h following admission. 64 (18.1%) patients did not receive any antiviral drug, while 174 (49.1%) patients received antiviral treatment with Lopinavir/ritonavir 400/100 mg twice daily. 22 (5.2%) patients received Tocilizumab 8 mg/kg from 13 March 2020.

**Table 2:** Treatments and outcomes of patients.

	N = 426
**Treatments**	
Lopinavir/ritonavir	174/354 (49.1%)
Hydroxychloroquine	249/354 (70.3%)
Corticosteroids	70/349 (20.0%)
Antibiotics	304/358 (84.9%)
Tocilizumab	22 (5.2%)
**Outcomes**	
Death	141 (33.1%)
Median time from symptoms onset to death (days)	11 (3–19)
Median time from hospitalization to death (days)	6 (3–11)
**Admission to ICU**	41 (9.6%)
Median time from symptoms onset to ICU admission (days)	11 (8–13)
Median time from hospitalization to ICU admission (days)	4 (2–6)
**Discharge**	239 (56.1%)
Median time from symptoms onset to discharge (days)	19 (9–24)
Median time from hospitalization to discharge (days)	10 (5–16)
Respiratory failure	245 (57.5%)
Acute kidney injury	26 (6.1%)
Acute cardiac injury	14 (3.3%)
Septic shock	7 (1.6%)
Thromboembolic events	7 (1.6%)

Data are expressed as median (interquartile range) or n (%).

### Clinical outcomes

On 21 April 2020, 141 (33.1%) patients had died. The median time from symptoms onset to death and from hospitalisation to death was 11 days (IQR 3–19) and 6 days (IQR 3–11), respectively. 41 (9.6%) patients had been transferred to ICU. The median time from hospitalisation to ICU admission was 4 days (IQR 2–6). 239 (56%) patients had been discharged and 46 (10.7%) patients were still hospitalised (17 of whom were still in ICU). Median time from hospitalisation to discharge was 10 days (IQR 5–18). The outcomes of patients who were still hospitalised have been updated as of 30 May, 2020: among these 46 patients, 5 (10.9%) had died, 8 (17.4%) were still in ICU, 12 (26.1%) were transferred to lower intensity care units and 21 (45.7%) were discharged.

Patients who died were older, had higher CCI and higher mEI score, higher CRP and LDH levels and lower lymphocyte count compared to survivor patients (Table 1). Hydroxycloroquine and antibiotics were used more frequently in patients who died compared to those who did not. The frequency of complications, such as respiratory failure, acute kidney injury, acute cardiac injury and septic shock was significantly higher in patients who died as compared to survivors. (Table S1).

Outcomes according to CCI and mEI score are showed in Table S2-S3. Area Under the Curve (AUC) for in-hospital mortality prediction was 0.80 (0.75–0.83) for CCI and 0.81 (0.76–0.85) for mEI (p-value for comparison = 0.468).

**Figure 1:**
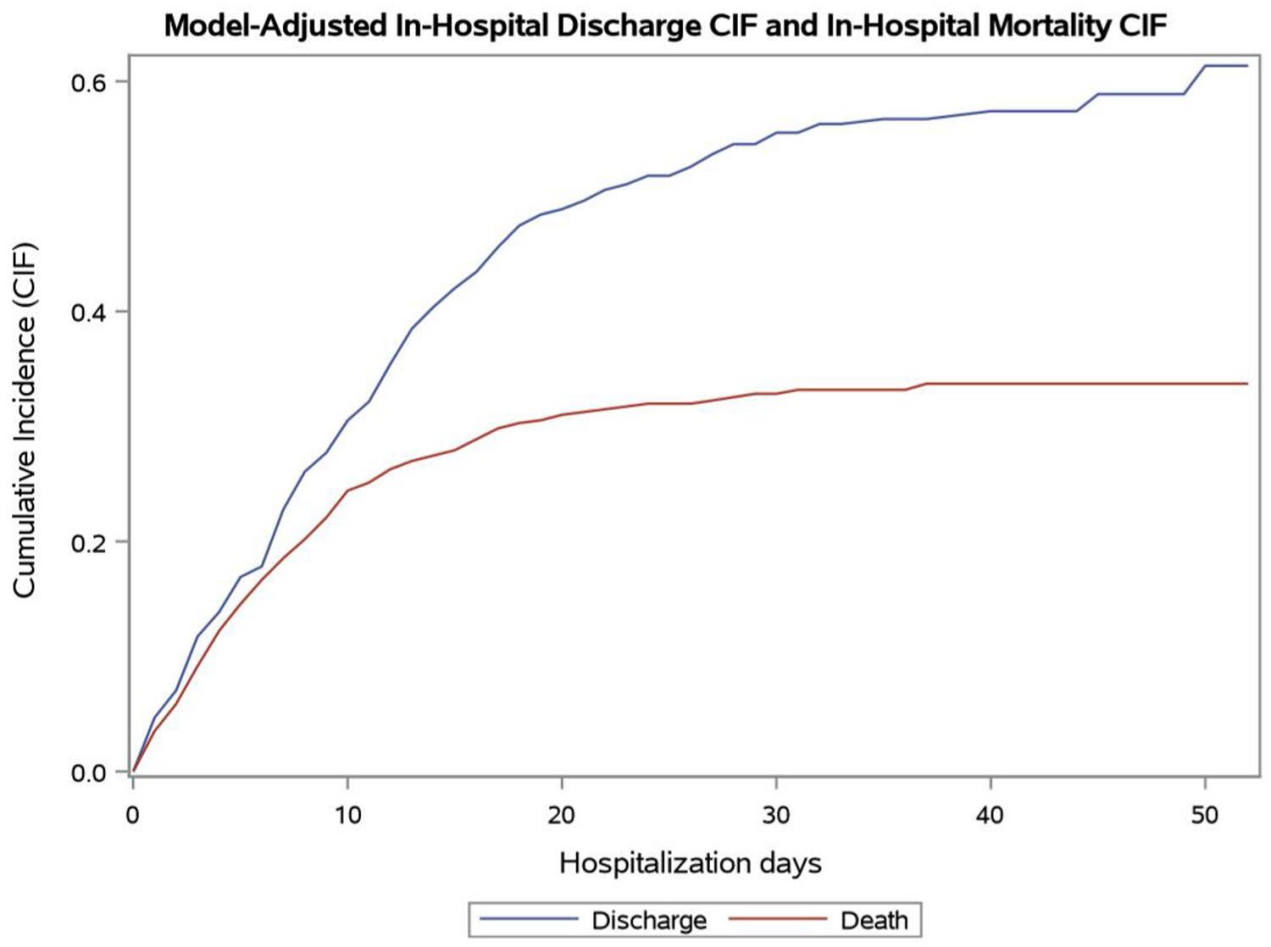
Cumulative incidence functions for in-hospital mortality and discharge of patients with Coronavirus Disease-19.

The CIF for in-hospital mortality is showed in Fig. 1. The estimated probability of in-hospital death was 24.4% during the first 10 days from hospitalization, 31.0% during the first 20 days and 33.7% at the end of follow-up. Univariate analysis for in-hospital mortality is reported in Table S4. Using the Fine and Gray model-to-model mortality, older age (70–79 years: HR 4.42, 95% CI 2.59–7.39, p < 0.0001. Over 79 years: HR 7.75, 95% CI 4.39–13.74, p < 0.0001), male sex (HR 1.85, 95%CI 1.22–2.89, p = 0.003), number of comorbidities higher than 3 (HR 3.63, p = 0.03), and time of hospital admission (between 4 and 16 March: HR 2.32, 95%CI 1.45–3.71, p = 0.001; between 17 and 30 March: HR 1.68, 95%CI 1.03–2.75, p = 0.04) were independently associated with higher in-hospital mortality, while time to ICU admission longer than 7 days (HR 0.19, 95%CI 0.05–0.67, p = 0.01) were independently associated with lower in-hospital mortality (Table 3). The CIFs for in-hospital mortality performed using the parameter estimates of the Fine and Gray model for each of these covariates are showed in Figures S1–S4. We also performed a multivariate model including single comorbidities, showing similar results (Table S5).

**Table 3:** Competing risk analysis by fine and gray model for in-hospital mortality and discharge.

	Fine and grey model							
	In-hospital mortality				Discharge			
	Beta	Standard error	Hazard ratio	p value	Beta	Standard error	Hazard ratio	p value
Age 70–79 years	1.49	0.27	4.42	<0.0001	− 0.95	0.19	0.39	<0.0001
Age > 80 years	2.05	0.29	7.75	<0.0001	− 1.31	0.25	0.27	<0.0001
Male sex	0.64	0.22	1.89	0.003	− 0.66	0.14	0.52	<0.0001
Number of comorbidities	0.52	0.25	3.63*	0.038	− 0.62	0.21	0.08**	0.003
Admission between March, 4 and March, 16	0.84	0.23	2.32	0.001	− 0.42	0.17	0.66	0.015
Admission between March, 17 and March, 30	0.52	0.25	1.68	0.048	− 0.39	0.16	0.68	0.017
Lymphocyte count, × 10^9^ per L	− 0.22	0.22	0.80	0.316	0.12	0.03	1.13	0.0001
Hydroxychloroquine	− 0.13	0.27	0.88	0.639	− 0.27	0.16	0.76	0.760
No ICU admission	0.34	0.45	1.41	0.449	Baseline			
Time to ICU admission lower than 3 days	Baseline				− 0.61	0.32	0.54	0.056
Time to ICU admission between 4 and 6 days	− 0.61	0.63	0.54	0.330	− 0.02	0.27	0.98	0.955
Time to ICU admission > 7 days	− 1.67	0.65	0.19	0.010	− 0.01	0.01	0.99	0.996

*Three comorbidities or more versus no comorbidities. Hazard ratio was 2.36 for two comorbidities versus no comorbidities and 1.54 for one comorbidity versus no comorbidity.

**Three comorbidities or more versus no comorbidities. Hazard ratio was 0.19 for two comorbidities versus no comorbidities and 0.44 for one comorbidity versus no comorbidity.

**Figure 2:**
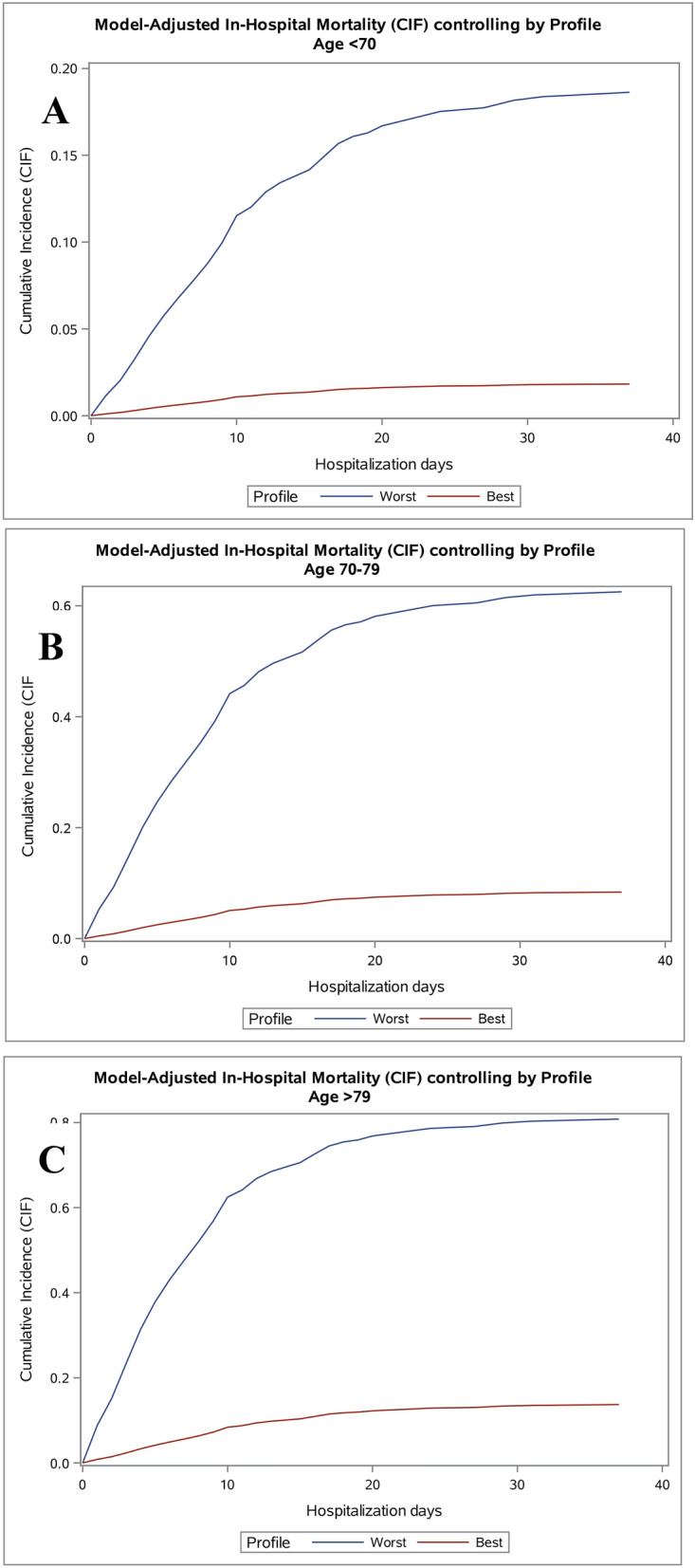
Cumulative incidence functions for in-hospital mortality performed using the parameter estimates of the Fine and Gray model and considering the best patient profile (female sex, number of comorbidities lower than 3 admitted between 21 February to 3 March 2020) and the worst patient profile (male sex, number of comorbidities higher than 3, admitted between 4 and 16 March 2020) according to age groups. **(A)** Age lower than 70 years. **(B)** Age between 70 and 79 years. **(C)** Age higher than 79 years.

These risk factors were then used to construct a model encompassing all patients grouped into a “best” and a “worst” class according to the presence or not of these factors. CIFs for the best class (female patients with less than 3 comorbidities, admitted between February, 21 and March, 3) and for the worst class (male patients with more than 3 comorbidities, hospitalized between 4 and 16 March) stratified by age group are showed in Fig. 2. At the end of follow-up, the probability of in-hospital death in patients younger than 70 years was 1.8% in the best class and 18.6% in the worst class. In patients with 70–79 years, the probability of in-hospital death at the end of follow-up was 8.3% in the best class and 62.5% in the worst class. In patients older than 80 years, the probability of in-hospital death at the end of follow-up was 13.7% in the best class and 80.8% in the worst class.

The characteristics and outcomes of patients according to the discharge status are reported in Tables S6–S7. The CIF for discharge is showed in Fig. 1. The estimated probability of discharge was 30.5% during the first 10 days from hospitalization, 48.8% during the first 20 days and 61.4% at the end of follow-up Univariate analysis is reported in Table S4. Tocilizumab use was significantly associated with a lower probability to be discharged at univariate analysis, however it was not included in the multivariate model because only 22 patients received Tocilizumab. Using the Fine and Gray model, we observed that lymphocytes count (HR 1.13, 95% CI 1.06–1.19, p = 0.0001) was independently associated with higher probability to be discharged, while older age (70–79 years: HR 0.39, 95%CI 0.27–0.55, p < 0.0001. Over 79 years: HR 0.27, 95% CI 0.16–0.44 p < 0.0001), number of comorbidities higher than 3 (HR 0.08, p < 0.0001), and time of hospital admission (between March, 4 and March, 16: HR 0.66, 95% CI 0.47–0.92, p = 0.02; between 17 and 30 March: HR 0.68, 95%CI 0.50–0.93, p = 0.02) were independently associated with lower probability to be discharged. (Table 3). The CIFs for discharge performed using the parameter estimates of the Fine and Gray model for each of these covariates are showed in Figures S5–S9. The CIFs for the best class and for the worst class according to age are showed in Figures S10–S12. At the end of follow-up, the probability of discharge in patients younger than 70 years was 99.5% in the best class and 31.5% in the worst class. In patients with 70–79 years, the probability of discharge at the end of follow-up was 84.6% in the best class and 12.6% in the worst class. In patients older than 80 years, the probability of discharge at the end of follow-up was 75.3% in the best class and 9.6% in the worst class.

## Discussion

This report, to our knowledge, is the first large retrospective study assessing competing risks in hospitalised patients with confirmed COVID-19 in Europe. Older age, male sex, comorbidities and hospital admission subsequent to March, 4 were significantly associated with a higher in-hospital death, by competing risk multivariate analysis.

When comparing our cohort with those described in the literature we noted that mortality was higher than that observed in other studies conducted both in and outside China^8–10^. The median age in our cohort was 68 years and 77 years in patients who died, which is higher than that observed in other studies. In-hospital mortality assessed by competing risks analysis was significantly higher in patients aged between 70 and 79 years and in those over 79, compared with patients younger than 70 years. By contrast, the probability of discharge was similar between patients of 70–79 years and those older than 79 years. The association between age and in-hospital mortality could be explained by the lower cardiopulmonary reserve, by the enhanced susceptibility to infections and by the inadequate control of anti-inflammatory mechanisms^11^.

In our cohort, the median Charlson comorbidity index was 3 and modified Elixhauser Index was 9.2. While the prevalence of comorbidities in our cohort was similar to that reported in the USA^10^, it was higher than that observed in Chinese cohorts^7,8^. Our results are in line with those of the Italian National Institute of Health, showing that approximately 61% of deceased Italian patients with COVID-19 had more than 3 comorbidities, while only 3.6% of patients who died had no comorbidity^12^. It is well known that COVID-19 patients with comorbidities are at high risk to develop a worst outcome. Several meta-analyses showed that comorbidities (specifically hypertension, respiratory system disease, cardiovascular disease, and chronic kidney disease) are associated with a higher risk of development of severe COVID-19^13–15^. Different comorbidity scores have been evaluated in COVID-19 patients, such as CCI^10^ and mEI score^16^. Our analysis showed that these two scores had a similar accuracy by AUC for the prediction of in-hospital death.

Male sex was an independent risk factor for in-hospital mortality and a lower probability of discharge. The association between gender and worst outcomes in COVID-19 is not fully understood. It has been proposed that female sex could be associated with a lower susceptibility to viral infections, with sex hormones playing a relevant role in innate and adaptive immune response^17^. A different expression of ACE 2 receptor has also been suggested as an explanation of the gender-associated mortality in COVID-19 patients^18^. Conversely, it has been suggested that males could be more prone to being affected by COVID-19 due to the higher smoking rate and higher prevalence of cardiovascular comorbidities^19^. However, our multivariate model suggested that sex was an independent predictor of mortality, and discharge regardless of comorbidities and evidence supporting smoking as a predisposing factor in men with COVID-19 are lacking. Unfortunately, we were unable to evaluate the association between smoking and clinical outcomes in COVID-19.

Patients who were admitted during the first weeks of the emergency had a significantly lower in-hospital mortality and a higher likelihood of discharge compared to those who were admitted during subsequent weeks, with the worst outcomes observed from 4 to 16 March 2020. One factor that many reports have addressed is the sequence of phases into which the disease has been divided, each corresponding to a different pattern of viral and immunological factors. Patient presentation in late phase may also have occurred, leading to the admission of an exceptionally large number of patients who needed hospitalisation in a short time span, resulting in a critical overload in the Policlinico San Matteo, in both triage and the management of the disease. These findings may be explained by also taking into consideration that during the first week many admissions were made for epidemiological reasons, leading to the hospitalisation of patients with few symptoms or mild disease.

Although ICU admission after 7 days from hospitalisation was independently and significantly associated with a lower risk of in-hospital mortality, the rapidity with which patients entered the ICU often concurrently with initiating other treatments makes the benefit of this treatment difficult to assess. Moreover, results from observational studies of drug effects should be interpreted with caution as they may be biased by survivor treatment selection bias, including time-related biases^20,21^.

In the literature, the use of composite endpoints (i.e. death or ICU admission) and, on the other hand, the implementation of traditional survival and Cox models are not appropriate in a disaster medicine setting such as that of COVID-19. The first assumption considers ICU and death to be equal, which is not true, while the traditional Cox model neglects to model discharge as an alternative endpoint. Competing risks analysis may provide further insights into the effect of interventions on the separate endpoint components^22^. We overcame this issue by performing a competing risks analysis taking into account two events (in-hospital death and discharge) and including ICU admission as a time-dependent covariate^23^. We suggest the use of a standardised methodology to assess treatment effects in observational studies in the complex clinical scenario of COVID-19. It should be underlined that COVID-19 case fatality ratio requires a dynamic assessment^24^ and that it decreased dramatically in Italy during the months that followed our study. This is could be to the improvements of the supportive treatments, as well as the general organization and bed occupancy. The competing risks model adopted is able to recognize effective and non effective predictors, as, for instance, our model excluded treatments since the very beginning. Nevertheless, we are aware that unknown risk factors are still incumbent in all the statistical analyses conducted till now, so frailty survival models can be applied in order to capture eventual and unknown source of variability. Summarising all the available evidence from randomised controlled trials and real-world comparative effectiveness studies, we are convinced that effective treatments for COVID-19 are still lacking and that therapies, such as specific antiviral drugs and immunomodulatory agents, remain an unmet and urgent medical need.

The main limitation of our study is the retrospective design. Retrospective studies have many problems that reduce their internal and external validity. When assessing retrospective cohort studies, the most important bias is the likelihood of the inappropriate selection of patients, which can lead to incorrect results and spurious associations. However, we included only consecutive patients with confirmed COVID-19, therefore we believe that selection bias was not relevant. Moreover, some potential confounders associated with the severity of COVID-19 (i.e. P/F ratio or circulating cytokine levels) and not available for this modelling could affect our results. Thus, we performed multivariate competing risks analysis to overcome this issue. Other limitations are the generalisability of our results to different populations and settings, particularly regarding the demographic structure of our country, including European elderly patients with a high prevalence of comorbidities. Finally, mortality was limited to in-hospital death, and discharged patients were assumed to still be alive during the study period.

In conclusion, our findings indicate that in a Lombardy cohort of elderly hospitalized patients, for the most part male with a high prevalence of comorbidities, COVID-19 is characterised by high in-hospital mortality. Older age, male sex, comorbidities and time of admission were found to be significant risk factors for in-hospital mortality and associated with a lower probability of being discharged.

## Methods

### Study setting

The SMatteo COvid19 Registry (SMACORE) is a cohort of patients with a confirmed diagnosis of COVID-19 disease referred to the IRCCS Policlinico San Matteo Hospital of Pavia, Italy from February 2020. The SMACORE database includes demographic, clinical laboratory tests, treatment, and outcome data. Ethics approval for observational research using SMACORE data was obtained from the local ethics committee.

This is a single centre, retrospective, observational cohort study and all patients of SMACORE cohort consecutively admitted to the Infectious Diseases Unit between 22 February and 30 March 2020, with a diagnosis of COVID-19 were enrolled. ICD-9 CM codes were reviewed, and clinical data were further extracted and reviewed by consulting the medical charts. Patients were followed until 21 April 21 2020. Laboratory confirmation of the SARS COV-2 infection was defined as positive Real-Time Reverse Transcriptase Polymerase Chain Reaction (RT-PCR) from clinical nasal swab.

### Statement

All methods were carried out in accordance with relevant guidelines and regulations and Ethics approval for observational research using SMACORE data was obtained from the local ethics committee and the informed consent has been obtained as by internal procedures.

### Data source

Demographic, clinical, laboratory, treatment, and outcome data were extracted from medical records using a standardised data collection form. The Charlson comorbidity index (CCI) and the modified Elixhauser index (mEi) were used to assess comorbidity^6,7^. CCI includes 16 comorbidities, predicting 10-year survival in patients with multiple comorbidities and was used as a measure of the total comorbidity burden. The mEi includes 11 comorbidities and it has been recently assessed in patients with COVID-19^16^. Imaging examinations were based on chest X-ray results. Although the benefits of a chest CT scan in achieving an early diagnosis of COVID-19 and in the follow-up of pneumonia evolution are well known^25^, we did not have the opportunity to include them in our clinical workout.

### Laboratory tests

Respiratory samples from the upper respiratory tract were prospectively collected and analysed at the Molecular Virology Unit, Fondazione IRCCS Policlinico San Matteo, Pavia, Italy, as part of the Regional SARS-CoV-2 surveillance and diagnosis plan in the Lombardy region. Total nucleic acids (DNA/RNA) were extracted from 200 ul of UTM using the QIAsymphon instrument with QIAsymphony DSP Virus/Pathogen Midi Kit (Complex 400 protocol) according to the manufacturer’s instructions (QIAGEN, Qiagen, Hilden, Germany). Specific RT-PCR targeting RNA-dependent RNA polymerase and E genes were used to detect the presence of SARS-CoV-2 in respiratory samples according to the WHO guidelines and published protocols^26,27^.

Routine blood examinations included complete blood count, serum creatinine, glutamic oxaloacetic transaminase (GOT) and glutamic pyruvic transaminase (GPT), lactate dehydrogenase (LDH), C-reactive protein (CRP) and troponin. Lymphocitopenia was defined as lymphocyte count < 1.5 × 10^9^/L. CRP was considered elevated above 10 mg/dL. LDH levels were considered elevated above 245 U/L. Blood cultures were performed in each patient and arterial-blood gas analysis (ABG) was performed when clinical signs of oxygen impairment were detected (e.g. tachypnoea and hypoxia).

### Treatment data

Treatment data included use of lopinavir/ritonavir, hydroxychloroquine, corticosteroids, tocilizumab and antibiotic drugs. Lopinavir/ritonavir 400/100 mg was administered orally twice daily for 14 days. Hydroxychloroquine (HCQ) 600 mg twice on day 1, then 400 mg daily for 7 days. Corticosteroid treatment consisted of dexamethasone 20 mg daily for 5 days in patients admitted from 22 February to 20 March and methylprednisolone 1 mg/kg intravenously daily for 5 days from 21 March to the end of follow-up. Tocilizumab 8 mg/kg was given intravenously in 1 or 2 doses from 13 March to the end of follow-up. A second dose was given 8–12 h after the first dose in patients with inadequate response. Antibiotic therapy consisted of a combination of piperacillin/tazobactam and doxycycline. Low (cannula and simple masks) and high (Venturi and reservoir masks, Nasal High Flow (NHF), helmet continuous positive airway pressure (CPAP)) flow oxygen support was provided when hypoxia was detected. Time to ICU admission was defined as the time from hospitalisation to ICU admission.

### Outcomes

The primary disease event was in-hospital mortality. Discharge was analysed as a competing event by competing risks analysis.

The criteria for discharge were absence of fever, clinical remission of respiratory symptoms, oxygen saturation greater than 94% and two nasal swab samples negative for SARS-CoV-2 RNA obtained at least 24 h apart.

Septic shock was defined according to the 2016 Third International Consensus Definition for Sepsis and Septic Shock^28^. Acute kidney injury was defined according to the KDIGO clinical practice guidelines^29^ and acute cardiac injury was diagnosed if serum levels of cardiac biomarkers (troponin) was above the 99th percentile upper reference limit, or if new abnormalities were shown in electrocardiography and echocardiography^30^.

### Statistical analysis

Data for continuous variables are presented as mean and standard deviation or median and interquartile ranges (IQR), and data for categorical variables are presented as frequency and percentage. Differences between continuous data were analysed by Student t test or by Mann–Whitney U test. Differences between categorical variables were analysed by χ^2^ test.

In-hospital mortality and discharge were evaluated by competing risks analysis, using cumulative incidence function (CIF)^5^. The proportional sub-distribution hazard model by Fine and Gray was fitted in order to estimate the effect of covariates on CIFs in-hospital death and discharge, including ICU admission as a time-dependent covariate^31^. Covariates used for multivariate analyses were chosen based on their significance in the univariate analysis (p < 0.10). Variables in the final model with a p-value < 0.05 were considered statistically significant. The results are expressed as adjusted hazard ratios (HR) and their 95% confidence intervals (CI). Discrimination of CCI and mEi for the prediction of in-hospital mortality was assessed by the area under the receiver operating characteristic curve (AUC). DeLong method was used to test whether the differences between AUCs were statistically significant^32^. Models used a complete-case analysis approach. Statistical analyses were completed in SAS version 9.4.

### Ethics approval

The study was approved by Fondazione IRCCS Policlinico San Matteo institutional review board for observational research using SMACORE data.

## Supplementary Information

Supplementary Figures.

Supplementary Tables.

Supplementary Legends.

## Data Availability

The authors agree to share relevant, anonymized data generated as part of the SMAtteo COvid19 REgistry (SMACORE) upon reasonable request.
