# Supplementary material for: Competing-risk analysis of coronavirus disease 2019 in-hospital mortality in a Northern Italian centre from SMAtteo COvid19 REgistry (SMACORE)
Source: Sci Rep. 2021 Jan 13;11:1137. doi: 10.1038/s41598-020-80679-2 (PMC7806993; doi:10.1038/s41598-020-80679-2)
Supplement: Supplementary file 1 — Supplementary Figures. [file 41598_2020_80679_MOESM1_ESM.pdf]

Figure S1.

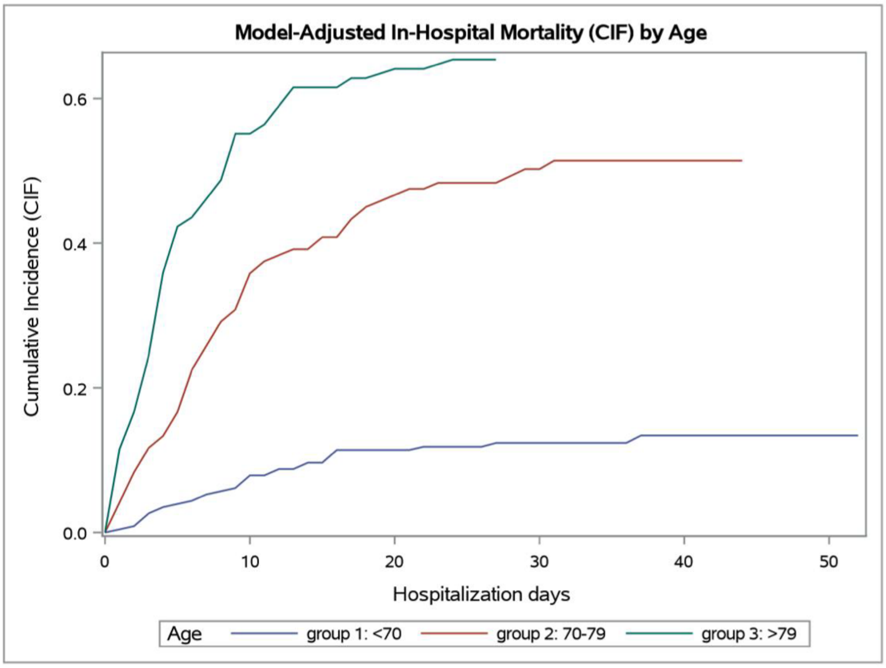
Model-Adjusted In-Hospital Mortality (CIF) by Age
Cumulative Incidence (CIF)
0.0
0.2
0.4
0.6
0
10
20
30
40
50
Hospitalization days
Age
group 1: <70
group 2: 70-79
group 3: >79

Figure S2

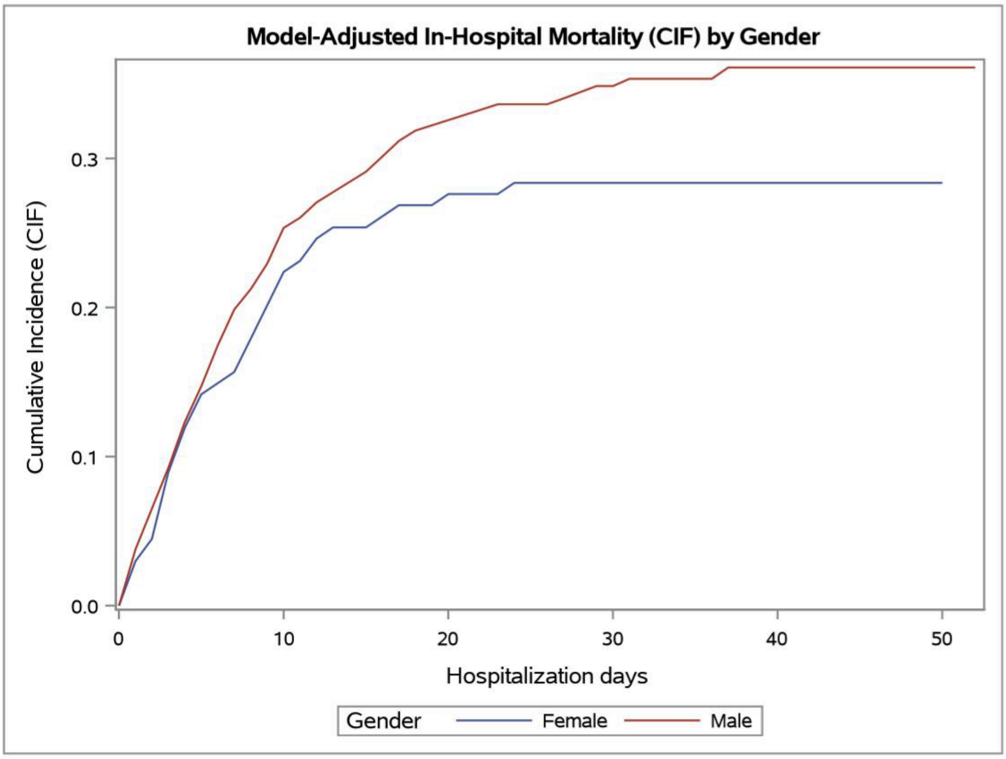
Model-Adjusted In-Hospital Mortality (CIF) by Gender
Cumulative Incidence (CIF)
0.0
0.1
0.2
0.3
0
10
20
30
40
50
Hospitalization days
Gender
Female
Male

Figure S3

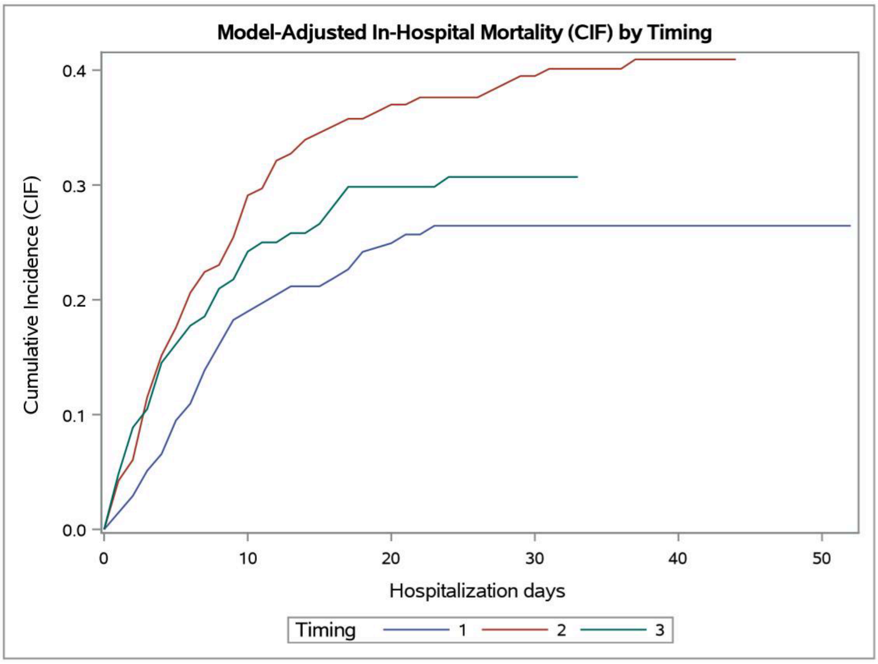

Model-Adjusted In-Hospital Mortality (CIF) by Timing
Cumulative Incidence (CIF)
0.0
0.1
0.2
0.3
0.4
0
10
20
30
40
50
Hospitalization days
Timing
1
2
3

Figure S4

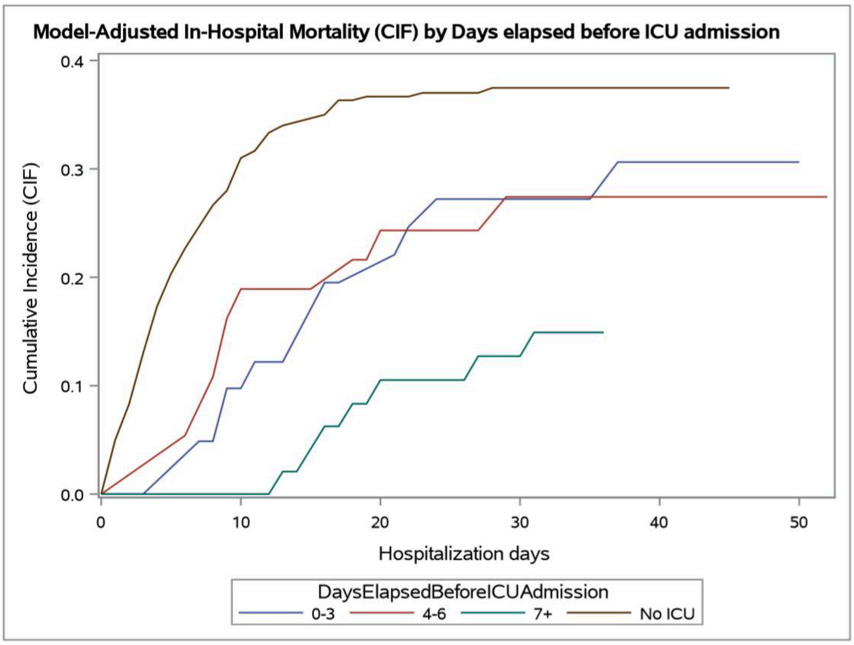
Model-Adjusted In-Hospital Mortality (CIF) by Days elapsed before ICU admission
Cumulative Incidence (CIF)
0.0
0.1
0.2
0.3
0.4
0
10
20
30
40
50
Hospitalization days
DaysElapsedBeforeICUAdmission
0-3
4-6
7+
No ICU

Figure S5

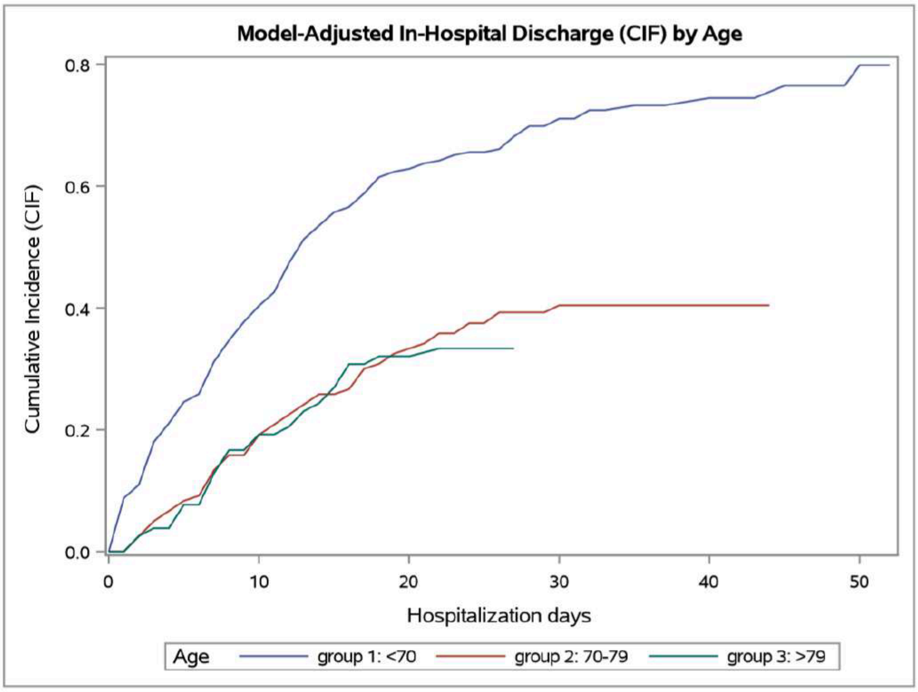

Model-Adjusted In-Hospital Discharge (CIF) by Age
Cumulative Incidence (CIF)
0.0
0.2
0.4
0.6
0.8
0
10
20
30
40
50
Hospitalization days
Age
group 1: <70
group 2: 70-79
group 3: >79

Figure S6

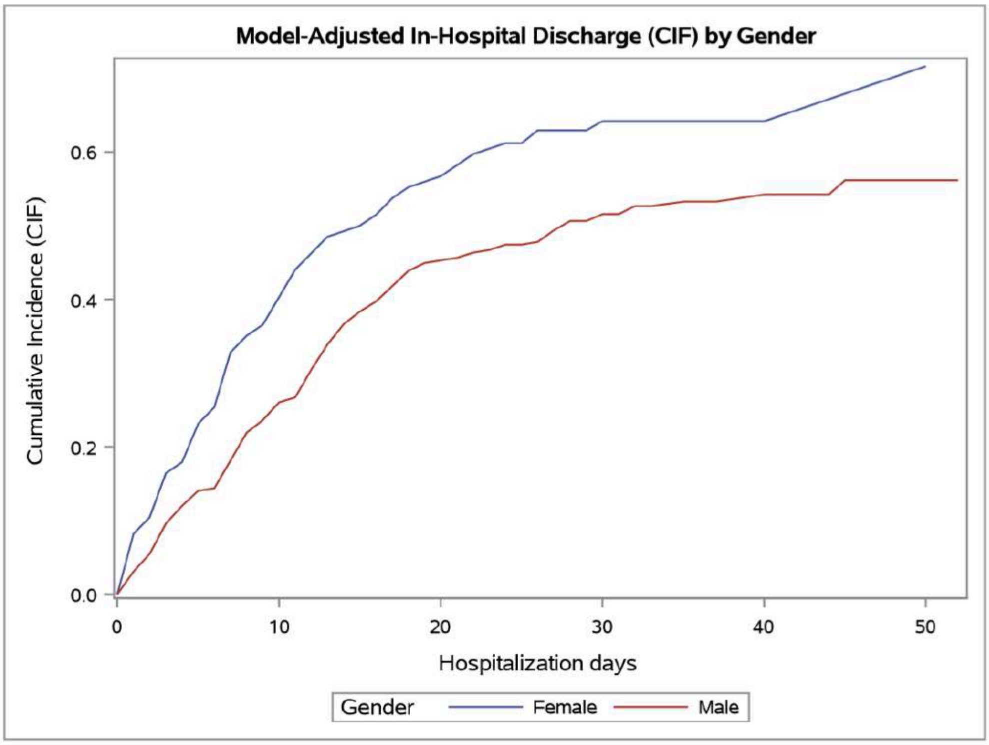

Model-Adjusted In-Hospital Discharge (CIF) by Gender
Cumulative Incidence (CIF)
0.0
0.2
0.4
0.6
0
10
20
30
40
50
Hospitalization days
Gender
Female
Male

Figure S7

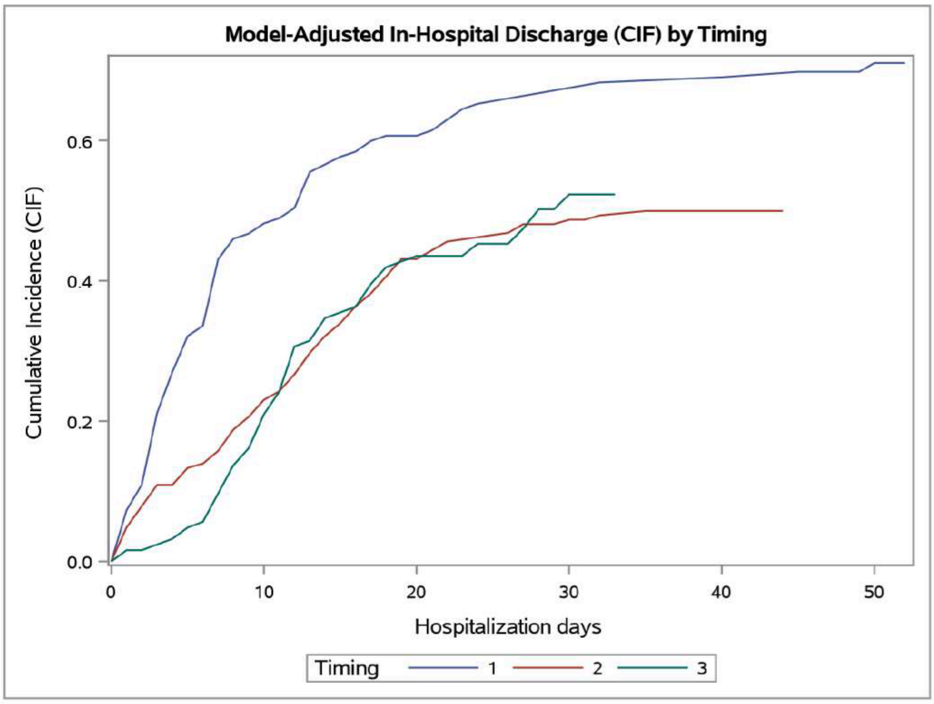

Model-Adjusted In-Hospital Discharge (CIF) by Timing
Cumulative Incidence (CIF)
0.0
0.2
0.4
0.6
0
10
20
30
40
50
Hospitalization days
Timing
1
2
3

Figure S8

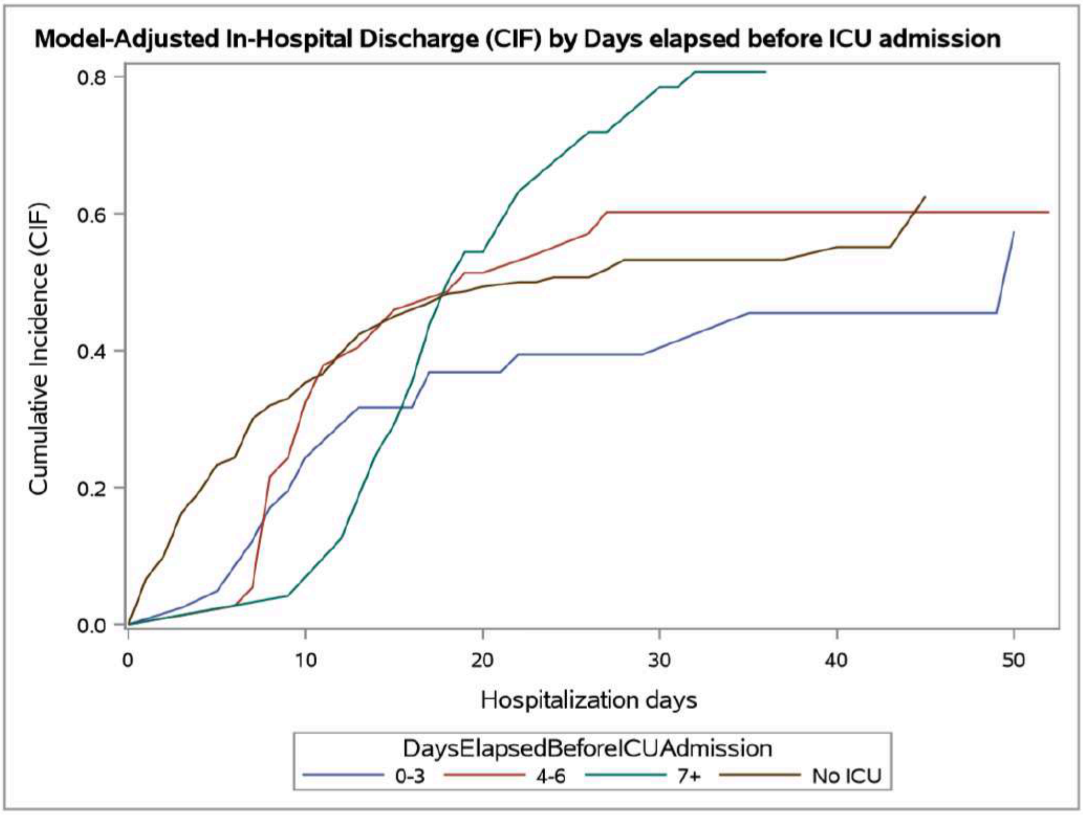

Model-Adjusted In-Hospital Discharge (CIF) by Days elapsed before ICU admission
0.8
0.6
0.4
0.2
0.0
Cumulative Incidence (CIF)
0
10
20
30
40
50
Hospitalization days
DaysElapsedBeforeICUAdmission
0-3
4-6
7+
No ICU

Figure S9

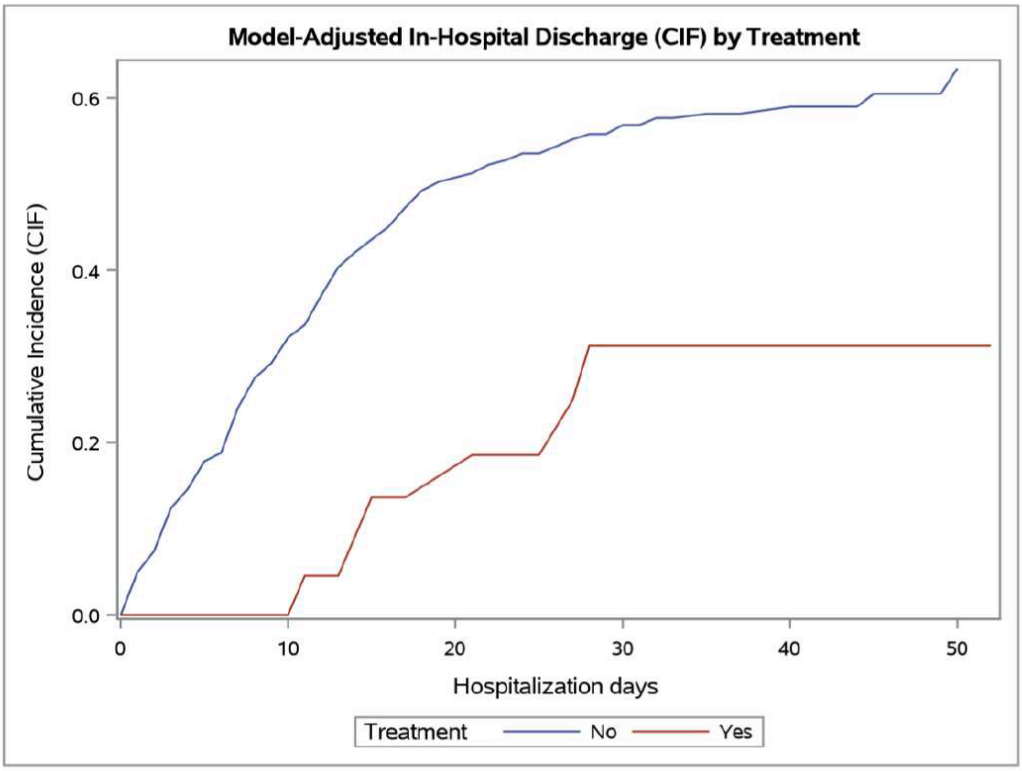

Model-Adjusted In-Hospital Discharge (CIF) by Treatment
Cumulative Incidence (CIF)
0.6
0.4
0.2
0.0
0
10
20
30
40
50
Hospitalization days
Treatment
No
Yes

Figure S10

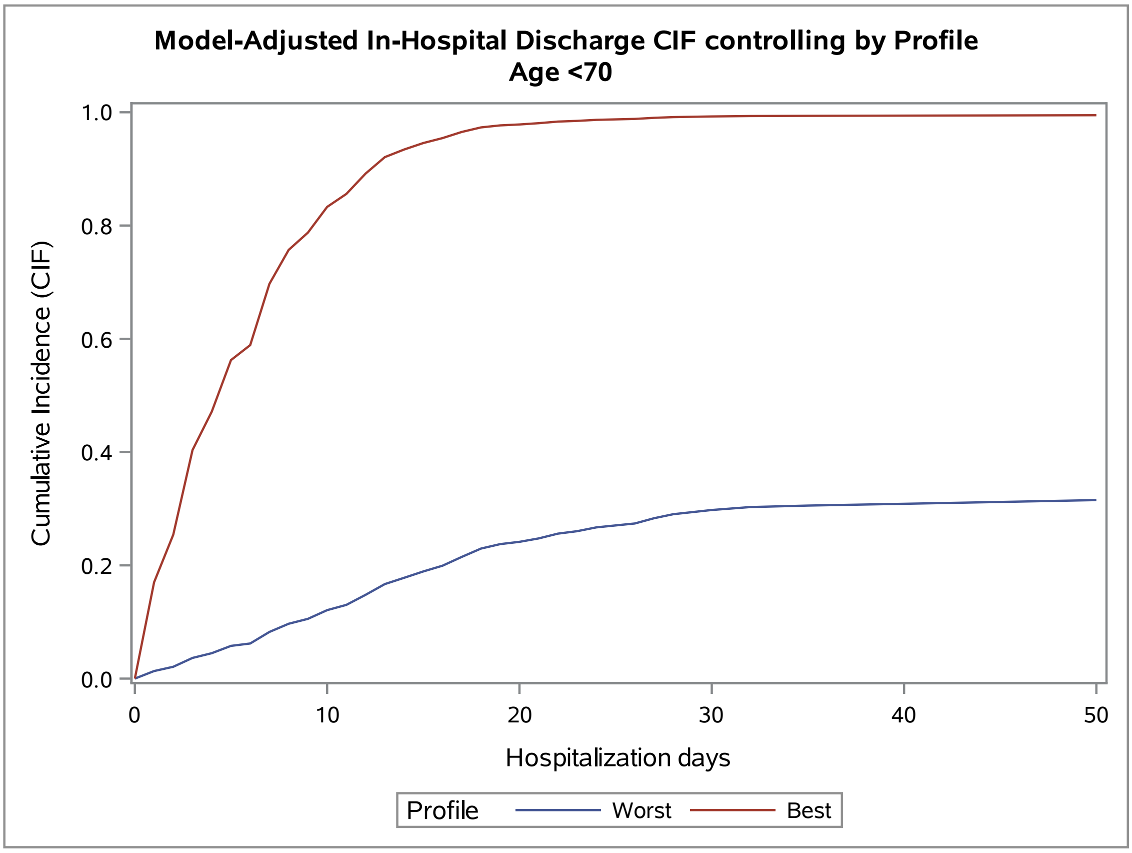
Model-Adjusted In-Hospital Discharge CIF controlling by Profile
Age <70
Cumulative Incidence (CIF)
1.0
0.8
0.6
0.4
0.2
0.0
0
10
20
30
40
50
Hospitalization days
Profile
Worst
Best

Figure S11

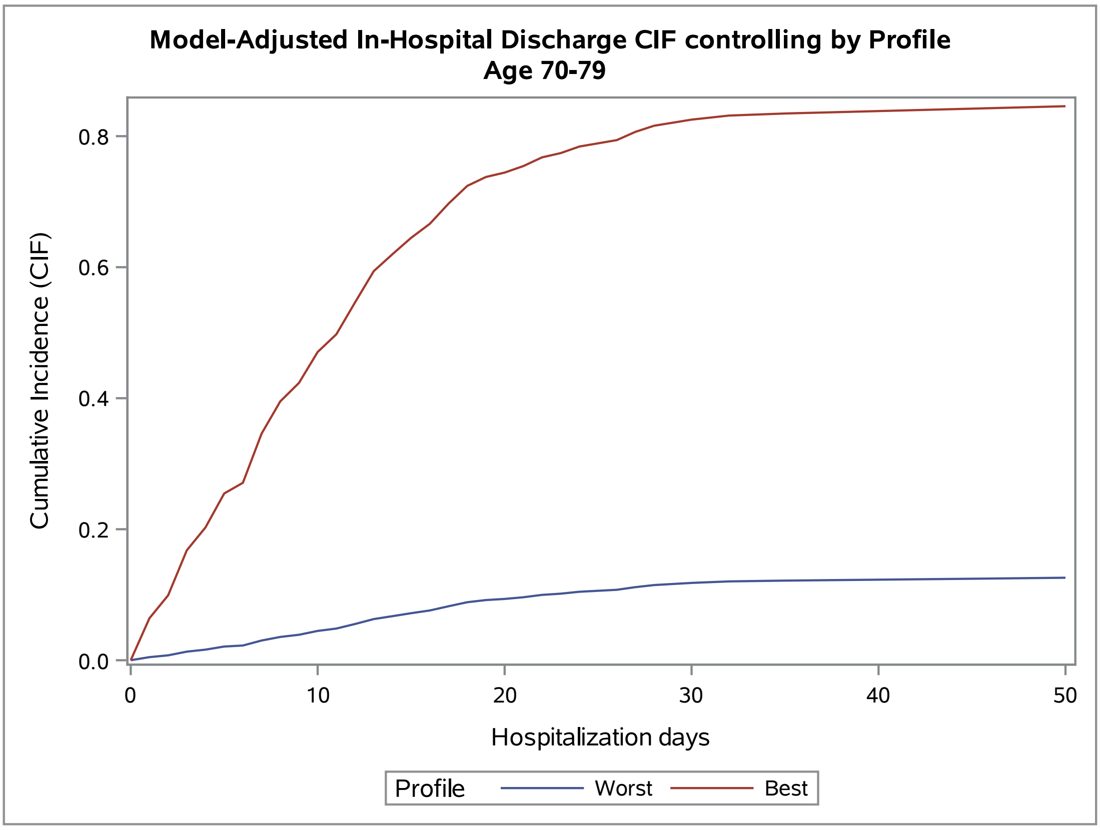
Model-Adjusted In-Hospital Discharge CIF controlling by Profile
Age 70-79
Cumulative Incidence (CIF)
0.0
0.2
0.4
0.6
0.8
0
10
20
30
40
50
Hospitalization days
Profile
Worst
Best

Figure S12

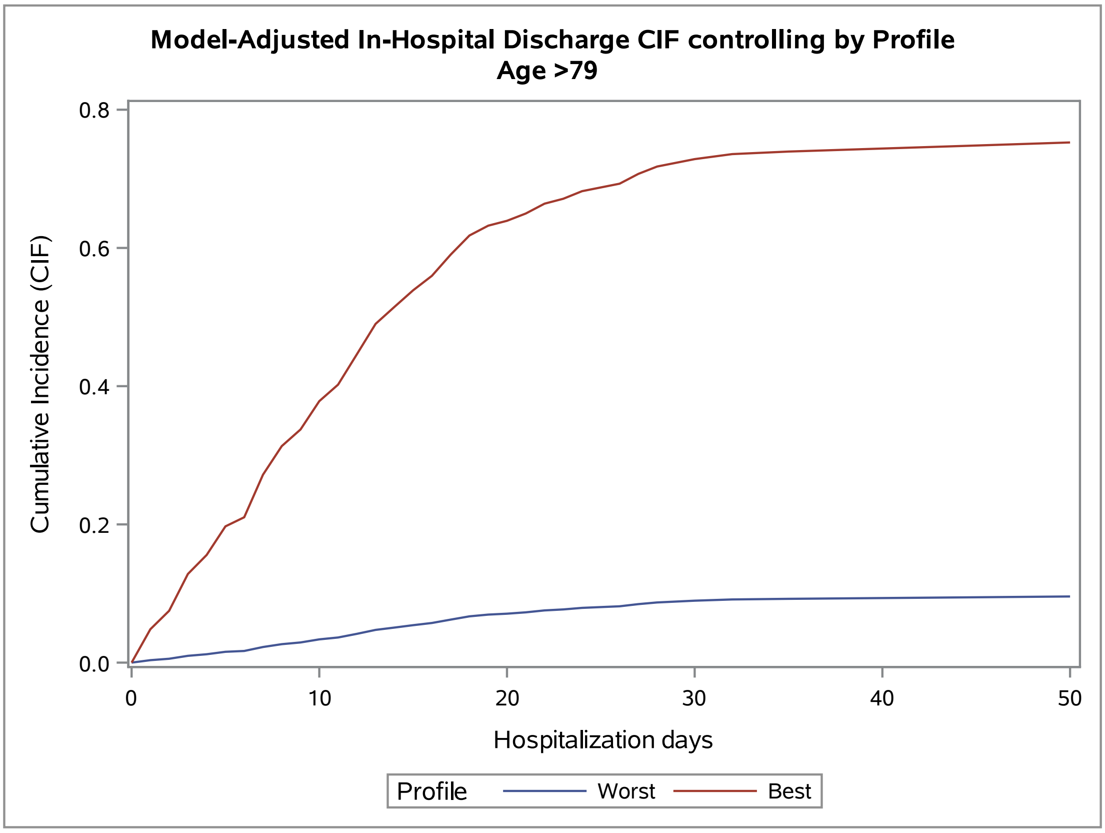

Model-Adjusted In-Hospital Discharge CIF controlling by Profile
Age >79
Cumulative Incidence (CIF)
0.0
0.2
0.4
0.6
0.8
0
10
20
30
40
50
Hospitalization days
Profile
Worst
Best
