# Supplementary material for: Competing-risk analysis of coronavirus disease 2019 in-hospital mortality in a Northern Italian centre from SMAtteo COvid19 REgistry (SMACORE)
Source: Sci Rep. 2021 Jan 13;11:1137. doi: 10.1038/s41598-020-80679-2 (PMC7806993; doi:10.1038/s41598-020-80679-2)
Supplement: Supplementary file 2 — Supplementary Tables. [file 41598_2020_80679_MOESM2_ESM.pdf]

Table S1. Treatments and outcomes of patients stratified according to death.

	Death (n=141)	Survivor (n=285)	*p*-value
**Treatments**			
Lopinavir/ritonavir	66/119 (55.5%)	108/235 (46.0%)	0.115
Hydroxychloroquine	92/119 (77.3%)	157/232 (67.7%)	0.079
Corticosteroids	21/119 (17.6%)	49/230 (21.3%)	0.504
Antibiotics	109/120 (90.8%)	195/238 (81.9%)	0.039
Tocilizumab	8 (5.7%)	14 (4.9%)	0.919
**Outcomes**			
Admission to ICU	22 (15.6%)	19 (6.7%)	0.006
Median time from hospitalization to ICU admission (days)	4 (2-6)	3 (2-6)	0.854
Respiratory Failure	119 (84.4%)	126 (44.2%)	<0.0001
Acute kidney injury	20 (14.2%)	6 (2.1%)	<0.0001
Acute cardiac injury	11 (7.8%)	3 (1.0%)	0.0007
Septic Shock	7 (4.9%)	0 (0)	0.0007
Thromboembolic events	2 (1.4%)	5 (1.8%)	0.882

Table S2 Outcomes of hospitalized COVID-19 patients according to Charlson Comorbidity Index.

	N=426	Charlson Comorbidity Index <3 (n=183)	Charlson Comorbidity Index ≥3 (n=243)	*p*-value
**Outcomes**				
Death	141 (33.1%)	19 (10.4%)	122 (50.2%)	<0.001
Median time from symptoms onset to death (days)	11 (3-19)	18 (11-21)	14 (11-18)	0.519
Median time from hospitalization to death (days)	6 (3-11)	10 (7-16)	6 (3-10)	0.014
Admission to ICU	41 (9.6%)	21 (11.4%)	20 (8.2%)	0.105
Median time from symptoms onset to ICU admission (days)	11 (8-13)	12 (6-15)	8 (6-11)	0.643
Median time from hospitalization to ICU admission (days)	4 (2-6)	3 (2-7)	5 (1-6)	0.691
Discharge	239 (56.1%)	141 (77.0%)	98 (40.3%)	<0.001
Median time from symptoms onset to discharge (days)	19 (9-24)	19 (13-26)	21 (16-25)	0.344
Median time from hospitalization to discharge (days)	10 (5-16)	8 (3-14)	12 (7-17)	0.004
Respiratory Failure	245 (57.5%)	87 (47.5%)	158 (65.0%)	<0.001
Acute kidney injury	26 (6.1%)	3 (1.6%)	23 (9.4%)	0.002
Acute cardiac injury	14 (3.3%)	2 (1.1%)	12 (4.9%)	0.083
Septic Shock	7 (1.6%)	3 (1.6%)	4 (1.6%)	0.711
Thromboembolic events	7 (1.6%)	4 (2.2%)	3 (1.2%)	0.687

Table S3. Outcomes of hospitalized COVID-19 patients according to modified Elixhauer score.

	N=426	Modified Elixhauer score < 9 (n=213)	Modified Elixhauer score ≥9 (n=213)	*p*-value
**Outcomes**				
Death	141 (33.1%)	20 (9.3%)	121 (56.8%)	<0.001
Median time from symptoms onset to death (days)	11 (3-19)	19.5 (18-23)	14 (9.0-19)	0.068
Median time from hospitalization to death (days)	6 (3-11)	10 (4.5-13)	6 (3-10.5)	0.127
Admission to ICU	41 (9.6%)	29 (13.6%)	12 (5.6%)	0.229
Median time from symptoms onset to ICU admission (days)	11 (8-13)	13 (11-15)	6 (3.5-10)	0.050
Median time from hospitalization to ICU admission (days)	4 (2-6)	3 (2-5)	3.5 (2-5.5)	0.986
Discharge	239 (56.1%)	175 (82.2%)	64 (30.0%)	<0.001
Median time from symptoms onset to discharge (days)	19 (9-24)	19 (13-26)	21 (17.5-26)	0.431
Median time from hospitalization to discharge (days)	10 (5-16)	9 (4-15)	12 (6-17)	0.076
Respiratory Failure	245 (57.5%)	128 (60.0%)	117 (54.9%)	<0.001
Acute kidney injury	26 (6.1%)	10 (4.7%)	16 (7.5%)	0.027
Acute cardiac injury	14 (3.3%)	5 (2.3%)	9 (4.2%)	0.093
Septic Shock	7 (1.6%)	2 (0.1%)	5 (2.3%)	0.848
Thromboembolic events	7 (1.6%)	2 (0.1%)	5 (2.3%)	0.848

Table S4. Competing risk analysis by univariate Fine and Gray model for in-hospital mortality and discharge.

	Fine and Grey model							
	In-hospital mortality				Discharge			
	Beta	Standard Error	Hazard ratio	p value	Beta	Standard Error	Hazard Ratio	p value
Age 70-79 years	1.56	0.24	4.77	<0.0001	-0.91	0.17	0.40	<0.0001
Age > 80 years	2.06	0.25	7.83	<0.0001	-1.16	0.22	0.31	<0.0001
Male sex	0.30	0.27	1.35	0.142	-0.43	0.14	0.65	0.003
Number of comorbidities	0.41	0.07	1.50	<0.0001	-0.42	0.08	0.66	<0.0001
Hypertension	0.21	0.19	1.24	0.260	-0.19	0.14	0.82	0.165
Diabetes	0.43	0.23	1.54	0.062	-0.40	0.19	0.67	0.038
Atrial fibrillation	0.85	0.25	2.34	0.001	-0.75	0.29	0.47	0.011
Coronary heart disease	1.16	0.23	3.18	<0.0001	-1.23	0.35	0.29	0.001
Chronic heart failure	0.55	0.28	1.74	0.050	-0.64	0.38	0.53	0.093
Chronic kidney disease	1.11	0.29	3.02	0.001	-1.12	0.39	0.33	0.005
Admission between March, 4 and March, 16	0.55	0.22	1.74	0.012	-0.52	0.16	0.60	0.002
Admission between March, 17 and March, 30	0.21	0.25	1.24	0.399	-0.52	0.16	0.59	0.002
Time to ICU admission > 7 days	-0.60	0.45	0.55	0.183	0.23	0.21	1.26	0.266
No ICU admission	0.63	0.30	1.89	0.033	-0.01	0.20	0.99	0.987
Glutamic oxaloacetic transaminase, U/L	-0.01	0.01	1.00	0.559	0.01	0.01	1.00	0.909
Glutamic pyruvic transaminase, U/L	-0.01	0.01	1.00	0.753	-0.01	0.01	1.00	0.891
Log (Creatinine, mg/dL)	0.06	0.12	1.06	0.617	-0.07	0.12	0.93	0.527

Log (Lactate dehydrogenase, U/L)	0.09	0.24	1.09	0.720	-0.18	0.21	0.83	0.377
Log (White Cell Blood Count, × 10^9 per L)	-0.14	0.20	0.87	0.500	0.01	0.18	1.01	0.969
Log (Lymphocyte Count, × 10^9 per L)	-0.35	0.17	0.71	0.043	0.45	0.15	1.56	0.003
Log (Neutrophil Count, × 10^9 per L)	-0.01	0.15	0.99	0.940	-0.08	0.14	0.92	0.552
Platelet Count, × 10^9 per L	-0.31	0.20	0.73	0.119	0.29	0.17	1.33	0.097
Lopinavir/ritonavir	0.27	0.20	1.31	0.173	-0.22	0.14	0.80	0.127
Hydroxychloroquine	0.44	0.22	1.56	0.043	-0.34	0.15	0.71	0.027
Corticosteroids	-0.19	0.23	0.82	0.409	-0.17	0.15	0.84	0.256
Tocilizumab	-0.01	0.32	0.99	0.968	-1.05	0.38	0.35	0.005

Table S5 Competing risk analysis by Fine and Gray model for in-hospital mortality and discharge including comorbidities.

	Fine and Grey model							
	In-hospital mortality				Discharge			
	Beta	Standard Error	Hazard ratio	p value	Beta	Standard Error	Hazard Ratio	p value
Age	0.07	0.01	1.08	<0.0001	-0.02	0.004	0.98	<0.0001
Male sex	0.52	0.21	1.68	0.012	-0.41	0.14	0.66	0.003
Diabetes	0.16	0.22	1.18	0.469	-0.30	0.20	0.74	0.137
Atrial fibrillation	0.04	0.26	1.04	0.871	0.08	0.31	1.09	0.785
Coronary heart disease	0.44	0.24	1.56	0.059	-0.46	0.35	0.63	0.193
Chronic heart failure	-0.30	0.31	0.74	0.336	-0.07	0.40	0.94	0.870
Chronic kidney disease	0.31	0.29	1.37	0.281	-0.65	0.42	0.52	0.126
Admission between March, 4 and March, 16	0.01	0.18	1.01	0.939	-0.20	0.14	0.82	0.163
Lymphocyte Count, × 10^9 per L	-0.20	0.24	0.82	0.399	0.10	0.04	1.10	0.030
Hydroxychloroquine	-0.05	0.19	0.95	0.787	-0.17	0.14	0.84	0.228
Time to ICU admission	0.06	0.04	1.06	0.224	-0.96	0.27	0.38	0.001

Table S6. Demographic, Clinical and Laboratory Characteristics of patients who have been discharged.

	Discharged (n=239)	Non-discharged (n=187)*	*p*-values
Age (years)	61.0 (48.0-73.0)	73.0 (65.5-80.0)	<0.0001
<50	63 (26.4%)	9 (4.8%)	<0.0001
50-59	45 (18.8%)	19 (10.2%)	0.019

60-69	57 (23.8%)	38 (20.3%)	0.453
70-79	50 (20.9%)	74 (39.6%)	<0.0001
>80	24 (10.0%)	47 (25.1%)	0.0001
Male sex	153 (64.0%)	139 (74.3%)	0.030
Comorbidity	130 (54.4%)	149 (79.7%)	<0.0001
Hypertension	74 (31.0%)	67 (35.8%)	0.406
Diabetes	29 (12.1%)	35 (18.7%)	0.110
Atrial fibrillation	12 (5.0%)	27 (14.4%)	0.003
Coronary Heart Disease	9 (3.8%)	32 (17.1%)	<0.0001
Obesity	13 (5.4%)	13 (7.0%)	0.718
Chronic kidney disease	6 (2.6%)	21 (11.2%)	0.001
Chronic heart failure	7 (2.9%)	16 (8.5%)	0.036
Chronic Liver disease	8 (3.3%)	14 (7.5%)	0.106
Chronic obstructive lung disease	9 (3.8%)	11 (5.9%)	0.396
History of malignancy	12 (5.0%)	5 (2.7%)	0.416
Active malignancy	6 (2.6%)	10 (5.3%)	0.214
Dementia	2 (0.9%)	12 (6.4%)	0.006
Charlson comorbidity index	2 (1-3)	4 (3-5)	<0.0001
Time from symptoms onset to hospitalization	8 (4-11)	7 (4-10)	0.117
Pneumonia at chest X-ray	167 (69.9%)	134 (71.6%)	0.769
Glutamic oxaloacetic transaminase, U/L	39 (27-55)	44 (31-69)	0.021
Glutamic pyruvic transaminase, U/L	30 (20-43)	36 (23-57)	0.012
C-reactive protein, mg/dL	7.64 (3.59-14.51)	10.4 (5.45-14.97)	0.017
Creatinine, mg/dL	0.87 (0.83-0.91)	0.91 (0.75-1.19)	0.029
Lactate dehydrogenase, U/L	365 (278-445)	380 (329-451)	0.033
Troponine, ng/L	36 (10-109)	21 (10-101)	0.324
White Cell Blood Count, × 10^9 per L	6.6 (5.4-9.1)	6.9 (5.1-9.2)	0.904

Lymphocyte Count, × 10^9 per L	0.8 (0.65-1.09)	0.74 (0.60-0.92)	0.067
Neutrophil Count, × 10^9 per L	5.07 (3.93-7.68)	5.55 (3.72-8.00)	0.478
Platelet Count, × 10^9 per L	206 (154-296)	202 (144-266)	0.199

*Non-discharged patients include 46 patients still hospitalized at the end of follow-up.

Table S7. Treatments and outcomes of patients who have been discharged.

	Discharged (n=239)	Non-discharged (n=187)*	*p*-value
**Treatments**			
Lopinavir/ritonavir	103/210 (49.0%)	71/144 (49.3%)	0.951
Hydroxychloroquine	144/209 (68.9%)	105/142 (73.9%)	0.367
Corticosteroids	42/208 (20.2%)	28/141 (19.9%)	0.952
Antibiotics	182/215 (84.7%)	122/143 (85.3%)	0.983
Tocilizumab	6 (2.5%)	16 (8.6%)	0.010
Low-molecular-weight heparin	34/112 (30.4%)	45/64 (70.3%)	<0.0001
**Outcomes**			
Median duration of hospitalization (days)	10 (5-18)	9 (4-25)	0.216
Respiratory Failure	122 (51.0%)	123 (65.8%)	0.003
Acute kidney injury	5 (2.1%)	21 (11.2%)	0.0002
Septic Shock	0	7 (3.7%)	0.009
Thromboembolic events	5 (2.1%)	2 (1.1%)	0.660
Acute cardiac injury	3 (1.3%)	11 (5.9%)	0.017

Data are expressed as median (interquartile range) or n (%).
