# Supplementary material for: Competing-risk analysis of coronavirus disease 2019 in-hospital mortality in a Northern Italian centre from SMAtteo COvid19 REgistry (SMACORE)
Source: Sci Rep. 2021 Jan 13;11:1137. doi: 10.1038/s41598-020-80679-2 (PMC7806993; doi:10.1038/s41598-020-80679-2)
Supplement: Supplementary file 3 — Supplementary Legends. [file 41598_2020_80679_MOESM3_ESM.docx]

**Figure S1.** Cumulative incidence functions for in-hospital mortality performed using the parameter

estimates of the Fine and Gray model according to age.

**Figure S2.** Cumulative incidence functions for in-hospital mortality performed using the parameter

estimates of the Fine and Gray model according to sex.

**Figure S3.** Cumulative incidence functions for in-hospital mortality performed using the parameter

estimates of the Fine and Gray model according to time of hospital admission (1: from 21 February to

3 March 2020. 2: From 4 March to 16 March 2020. 3: From 17 March to 30 March 2020).

**Figure S4.** Cumulative incidence functions for in-hospital mortality performed using the parameter

estimates of the Fine and Gray model according to time to ICU admission.

**Figure S5.** Cumulative incidence functions for discharge performed using the parameter estimates of the Fine and Gray model according to age.

**Figure S6.** Cumulative incidence functions for discharge performed using the parameter estimates of the Fine and Gray model according to sex.

**Figure S7.** Cumulative incidence functions for discharge performed using the parameter estimates of the Fine and Gray model according to time of hospital admission (1: from 21 February to 3 March 2020. 2: From 4 March to 16 March 2020. 3: From 17 March to 30 March 2020).

**Figure S8.** Cumulative incidence functions for discharge performed using the parameter estimates of the

Fine and Gray model according to time to ICU admission.

**Figure S9.** Cumulative incidence functions for discharge performed using the parameter estimates of the

Fine and Gray model according to Tocilizumab treatment.

**Figure S10.** Cumulative incidence functions for discharge performed using the parameter estimates of the

Fine and Gray model and considering the best patient profile (female sex, number of comorbidities lower

than 3 admitted between 21 February to 3 March 2020) and the worst patient profile (male sex, number of

comorbidities higher than 3, admitted between 4 March, 4 and 16 March 2020) according to age lower than 70 years.

**Figure S11.** Cumulative incidence functions for discharge performed using the parameter estimates of the

Fine and Gray model and considering the best patient profile (female sex, number of comorbidities lower

than 3 admitted between 21 February to 3 March 2020) and the worst patient profile (male sex, number of

comorbidities higher than 3, admitted between 4 March and 16 March 2020) according to age between 70 and 79 years.

**Figure** S12. Cumulative incidence functions for discharge performed using the parameter estimates of the

Fine and Gray model and considering the best patient profile (female sex, number of comorbidities lower

than 3 admitted between 21 February to 3 March 2020) and the worst patient profile (male sex, number of

comorbidities higher than 3, admitted between 4 March and 16 March 2020) according to age higher than 79

years.
